# GDNF Gene Therapy to Repair the Injured Peripheral Nerve

**DOI:** 10.3389/fbioe.2020.583184

**Published:** 2020-10-30

**Authors:** Ruben Eggers, Fred de Winter, Martijn R. Tannemaat, Martijn J. A. Malessy, Joost Verhaagen

**Affiliations:** ^1^Laboratory for Neuroregeneration, Netherlands Institute for Neuroscience, Institute of the Royal Academy of Arts and Sciences, Amsterdam, Netherlands; ^2^Department of Neurology, Leiden University Medical Center, Leiden, Netherlands; ^3^Department of Neurosurgery, Leiden University Medical Center, Leiden, Netherlands; ^4^Department of Molecular and Cellular Neurobiology, Center for Neurogenomics and Cognition Research, Vrije Universiteit Amsterdam, Amsterdam, Netherlands

**Keywords:** gene therapy, peripheral nerve injury, nerve regeneration, ventral root avulsion, axonal regeneration

## Abstract

A spinal root avulsion is the most severe proximal peripheral nerve lesion possible. Avulsion of ventral root filaments disconnects spinal motoneurons from their target muscles, resulting in complete paralysis. In patients that undergo brachial plexus nerve repair, axonal regeneration is a slow process. It takes months or even years to bridge the distance from the lesion site to the distal targets located in the forearm. Following ventral root avulsion, without additional pharmacological or surgical treatments, progressive death of motoneurons occurs within 2 weeks (Koliatsos et al., 1994). Reimplantation of the avulsed ventral root or peripheral nerve graft can act as a conduit for regenerating axons and increases motoneuron survival (Chai et al., 2000). However, this beneficial effect is transient. Combined with protracted and poor long-distance axonal regeneration, this results in permanent function loss. To overcome motoneuron death and improve functional recovery, several promising intervention strategies are being developed. Here, we focus on GDNF gene-therapy. We first introduce the experimental ventral root avulsion model and discuss its value as a proxy to study clinical neurotmetic nerve lesions. Second, we discuss our recent studies showing that GDNF gene-therapy is a powerful strategy to promote long-term motoneuron survival and improve function when target muscle reinnervation occurs within a critical post-lesion period. Based upon these observations, we discuss the influence of timing of the intervention, and of the duration, concentration and location of GDNF delivery on functional outcome. Finally, we provide a perspective on future research directions to realize functional recovery using gene therapy.

## Introduction

Root avulsion lesions are typically part of a brachial plexus traction injury which occurs during traffic accidents and complicated childbirth. Following an avulsion lesion, the rupture of nerve root filaments from the surface of the spinal cord leads to a combined central and peripheral nervous system lesion. This lesion is often not limited to only one nerve root, but consists of the avulsion of multiple roots. Despite neurosurgical repair, the degree of recovery of function in patients suffering a brachial plexus lesion often remains poor and results in lifelong dysfunction and pain. Thus, in order to regain useful function following neurosurgical repair, multiple supplementary regenerative treatment strategies are required. Here, we will focus on GDNF gene therapy while other intervention strategies have been discussed elsewhere (Chu and Wu, 2009; Carlstedt, 2010; Eggers et al., 2016).

## The Value of the Ventral Root Avulsion as a Model to Study Neurotmetic Nerve Lesions and Treatment Strategies

A ventral root avulsion lesion is not frequently used as a lesion model to study axonal regeneration. This might partially be due to the complexity of the lesion model and surgical procedures. Additionally, from a clinical perspective, this type of lesion might be considered beyond repair (Seddon, 1942; Robotti et al., 1995). Most often, studies on experimental peripheral nerve regeneration in mouse or rat models use either a crush (axonotmesis) or transection (neurotmesis) of a mixed peripheral nerve such as the sciatic, femoral, median or ulnar nerve. These lesions are performed relatively close to the target organ, which requires only a relatively short follow-up period. Following these types of lesions, spontaneous axonal regeneration and recovery of function is quite robust. These studies have provided important insights in the pathophysiological understanding of the regenerative response mechanisms in an injured peripheral nerve. However, translation to the clinical situation with different anatomical dimensions appears unsatisfactory. In patients, proximal lesions lead to a prolonged denervation period associated with a limited degree of axon regeneration and poor recovery of function. In order to obtain translatable data, animal models that more closely mimic the clinical situation are required. Long denervation and regeneration time-periods have been achieved by delaying surgical repair (Fu and Gordon, 1995; Gordon et al., 2011; Jonsson et al., 2013; Ronchi et al., 2017) or creating large nerve defects (Saheb-Al-Zamani et al., 2013; Marquardt et al., 2015; Hoben et al., 2018). In agreement with clinical observations, in these models of protracted denervation, spontaneous recovery of function is extremely poor or even absent. The main cause for the limited functional recovery in these injury models is attributed to the failure of repair Schwann cells to continue to support axon regeneration after a critical post-lesion period of 6 to 8 weeks (Sulaiman and Gordon, 2000; Hoke et al., 2002). After this period, a state of chronic denervation develops and the process of supported regeneration comes to a halt.

**FIGURE 1:**
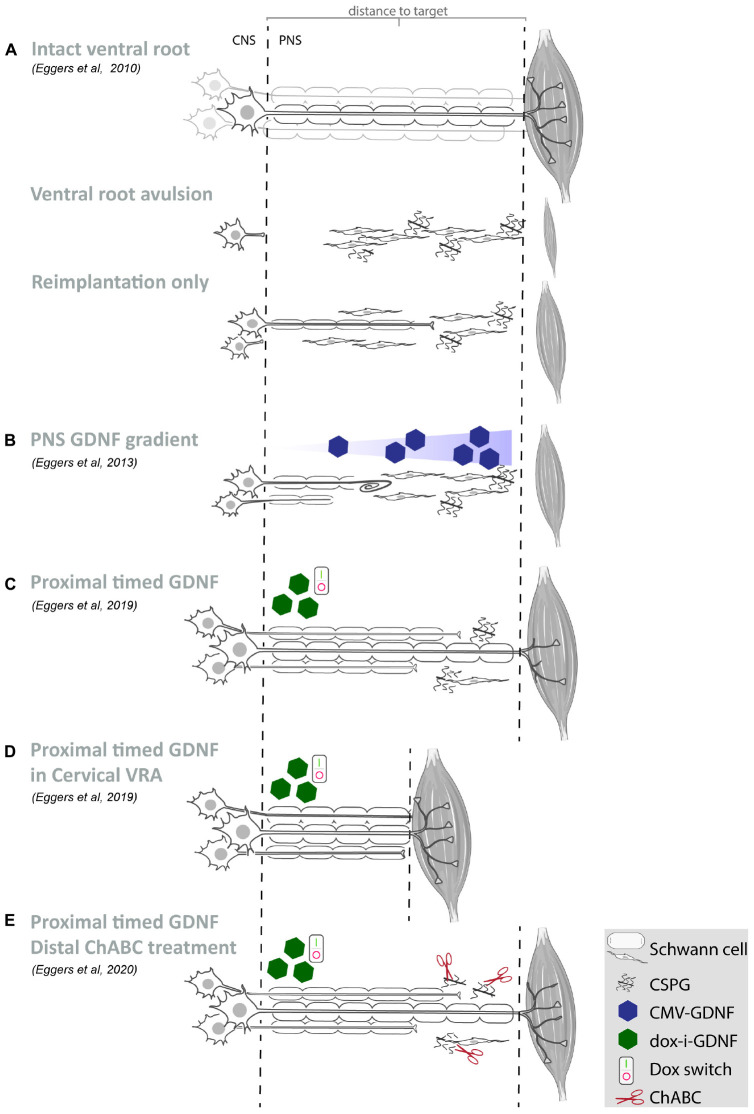
Summary of our recent GDNF treatment strategies. **(A–E)** Schematic overview of motoneurons located in the spinal ventral horn and axonal projections toward the distal target muscle. For each study, the experimental strategy and primary experimental outcomes are shown. Depicted are schematic representations of the degree of motoneuron loss, axonal outgrowth, muscle atrophy and target reinnervation based upon histological or electrophysiological observations. **(A)** Ventral root avulsion or reimplantation only leads to severe motoneuron loss and poor axonal regeneration. **(B)** An increasing GDNF gradient in the peripheral nerve resulted in coil formation already at relatively low GDNF concentrations and the degree of coil formation strongly correlates to the level of expression. Proximal timed GDNF treatment inside the reimplanted ventral root using a regulatable gene switch in a lumbar ventral root avulsion **(C)** or cervical ventral root avulsion **(D)** results in enhanced motoneuron survival and axonal outgrowth. The degree of muscle reinnervation and functional recovery is enhanced following short-distance **(D)** axonal regeneration. **(E)** As a first step toward improving distal axonal regeneration we combined proximal timed GDNF treatment and peripheral ChABC mediated CSPG digestion to overcome the inhibitory chronically denervated peripheral nerve environment. (VRA, ventral root avulsion; CNS, central nervous system; PNS, peripheral nervous system; CSPG, chondroitin sulfate proteoglycan; ChABC, chondroitinase ABC; GDNF, glial cell line-derived neurotrophic factor).

Reimplantation of the avulsed ventral root has been pioneered by the Carlstedt laboratory and in patients has resulted in recovery in the proximal limb similar to that achieved by conventional nerve grafts (Carlstedt et al., 1986, 1995, 2000; Htut et al., 2007). Reimplantation has since been used by several laboratories worldwide (Wu et al., 2003; Bergerot et al., 2004; Haninec et al., 2004; Hoang and Havton, 2006; Penas et al., 2011; Barbizan et al., 2013; Pajenda et al., 2014). Directly following avulsion progressive death of motoneurons occurs within 2 weeks (Koliatsos et al., 1994). Acute reimplantation of a peripheral nerve graft or avulsed ventral root enhances motoneuron survival and acts as a conduit for regenerating axons (Wu et al., 1994; Hoang and Havton, 2006; Ohlsson et al., 2006). However, a spatio-temporal analysis following the trajectory between the spinal motoneurons and distal target muscle after experimental lumbar ventral root avulsion and reimplantation, revealed that the beneficial effect of ventral root reimplantation on motoneuron survival is not maintained beyond 4 weeks (Figure 1A; Eggers et al., 2010). The initial axonal outgrowth response is robust, but includes aberrant growth to ectopic sites while the number of axons able to regenerate over a long distance is low. The failure to regenerate over long distances is associated with a loss of endogenous peripheral neurotrophic support, including decreasing levels of glial cell line-derived neurotrophic factor (GDNF) protein. Similar to chronic denervation models, after a ventral root avulsion lesion, the pro-regenerative period is limited to 6 to 8 weeks, after which the ability for long distance axon regeneration becomes increasingly poor (Eggers et al., 2010, 2019a; Torres-Espin et al., 2013).

The regeneration distance of 12 cm which is created with a lumbar ventral root avulsion in the rat is the longest peripheral nerve regeneration distance possible in small animal research. Performing a spinal root avulsion in larger species such as rabbit (Lang et al., 2005; Reichert et al., 2015), cat (Cullheim et al., 1989; Rafuse et al., 1992; Hoffmann et al., 1996), or macaque (Hallin et al., 1999; Ohlsson et al., 2013; Nieto et al., 2019) inherently results in longer regeneration distances. The proof of concept studies in these larger animals mainly focused on axonal regeneration into the reimplanted root, showing the feasibility of ventral root reimplantation as a clinical treatment option. Functionally, signs of reinnervation on electromyography and co-contractions due to axonal misrouting are primarily observed in the proximal musculature. Considering our and others’ observed degree of long-distance axonal regeneration and functional recovery following lumbar ventral root avulsion in the rat, in our view, performing regenerative gene therapy studies in larger animals is not warranted before meaningful functional recovery in the rat is achieved.

A ventral root avulsion lesion has several unique characteristics, which are distinct from most peripheral nerve regeneration models. *First*, avulsion is an extensive proximal nerve lesion, which combines a longitudinal spinal cord lesion with denervation of the complete peripheral nerve (Carlstedt and Havton, 2012). *Second*, axotomy close to the motoneuron cell body results in progressive degeneration and death of spinal motoneurons, which does not occur if axotomy is performed more than 4 mm distal from the cell body (Gu et al., 1997). *Third*, ventral root avulsion is a selective motor axon lesion, while the afferent sensory axons remain intact. *Fourth*, in delayed surgical repair models, a chronically denervated distal nerve is achieved by halting regeneration until a second additional repair surgery is performed allowing regeneration to proceed. This contrasts with acute root avulsion and reimplantation, which allows for uninterrupted axonal regeneration, while the required long regeneration distance leads to a prolonged period of distal Schwann cell denervation. Clinically, brachial plexus lesions can be variable consisting of a combined axonotmetic and neurotmetic nerve lesion of multiple adjacent motor and sensory nerve roots (Narakas, 1985). Although our selective ventral root avulsion and acute reimplantation does not reflect this heterogeneity, by performing a complete motor nerve lesion, this methodology provides us with a highly reproducible and predictable model for long-distance motor axon regeneration accompanied with chronic denervation. Compared to chronic peripheral nerve lesion models, an avulsion lesion more closely represents the severe pathogenesis as observed in the clinic after a proximal nerve lesion such as a brachial plexus injury.

## GDNF Gene Therapy as a Powerful Treatment Strategy

To improve recovery of function, supplementary regenerative treatment strategies are required. We and others have shown that Glial cell line-derived neurotrophic factor (GDNF) is a compelling treatment candidate due to its role in neuronal differentiation and identification as a potent motoneuron survival and axon outgrowth factor (Henderson et al., 1994; Li et al., 1995). Furthermore, in motoneurons following axotomy, the GDNF receptors c-RET and GFRα-1 are strongly upregulated (Hammarberg et al., 2000). In Schwann cells, GDNF-mediated signaling cascades play an important role in myelination, proliferation and migration (Iwase et al., 2005).

However, GDNF and other neurotrophic factors have a short half-life, exhibit poor tissue penetration and systemic or topical delivery of GDNF results in unwanted side effects in non-targeted tissues. Here, the advantage of gene therapy is the sustained production of GDNF protein by viral vector transduced cells, resulting in the constant availability of biologically active therapeutic protein restricted to the site of viral vector application. Despite these advantages, persistent high local levels of GDNF expression lead to impaired axon regeneration by inducing coil formation at the site of GDNF expression (Blits et al., 2004; Love et al., 2005; Eggers et al., 2008, 2013; Tannemaat et al., 2008; Santosa et al., 2013; Shakhbazau et al., 2013; Marquardt et al., 2015; Ee et al., 2017; Wang et al., 2018). Application of a 4 cm long increasing proximo-distal GDNF gradient in a lesioned peripheral nerve, demonstrated that an increasing GDNF concentration enhanced distal axonal sprouting and axon numbers (Eggers et al., 2013). However, coil formation was observed already at relatively low GDNF concentrations (Figure 1B) and the degree of coil formation strongly correlates to the level of expression. These observations indicate that it is important to control the timing, dose and location of neurotrophic factor expression in order to achieve successful long-distance axonal regeneration (Harvey et al., 2015).

In recent proof of concept studies, transplantation of engineered Schwann cells in the peripheral nerve allowed for doxycycline-inducible GDNF expression (Shakhbazau et al., 2013; Marquardt et al., 2015). In contrast to the disrupted axonal regeneration following persistent GDNF expression, time restricted GDNF expression was beneficial for axonal growth. These findings are in agreement with studies showing the paramount importance of achieving control over neurotrophic factor delivery (Kemp et al., 2011; Pajenda et al., 2014; Marquardt et al., 2015; Santos et al., 2016; Tajdaran et al., 2016). Although the doxycycline-inducible system is a robust platform for therapeutic gene regulation *in vivo*, in rodents and non-human primates long-term therapeutic gene regulation is hampered due to an immune response directed against the foreign rtTA transactivator, resulting in an immune-mediated removal of transduced cells (Favre et al., 2002; Ginhoux et al., 2004; Le Guiner et al., 2014). This compromises the experimental *in vivo* use and clinical translation.

To obtain sustained control over GDNF expression, an immune-evasive doxycycline-inducible GDNF gene switch (dox-i-GDNF) has been previously developed (Hoyng et al., 2014; Eggers et al., 2019b). Using this dox-i-GDNF system, we investigated whether time-restricted GDNF expression improves motoneuron survival and attenuates coil formation following a ventral root avulsion lesion. Injection into the reimplanted lumbar ventral root close to the motoneuron pool and a 4 week timed GDNF expression was sufficient to enhance motoneuron survival up to 45 weeks (Figures 1C–E). This achievement is clinically relevant because increased motoneuron survival significantly augments the chance of axonal outgrowth and extends the time window for long-distance regeneration. In contrast to persistent GDNF expression, time-restricted GDNF expression attenuated coil formation and leads to a two-fold increase in axonal outgrowth over a distance of 10 cm. This increased outgrowth facilitated an earlier and enhanced muscle reinnervation as shown by the improved electromyographical recovery in the distal denervated musculature (Figure 1C; Eggers et al., 2019b). Despite these promising results, the degree of recovery remained insufficient to enable voluntary hind paw function. The regenerating axons present in the distal sciatic nerve originate from only 8 to 10% of the surviving motoneurons, whereas the remaining surviving motoneurons were unable to project an axon toward and beyond a 10 cm distance from the spinal cord. Although it remains difficult to determine the threshold that needs to be overcome to obtain functional recovery, it has been suggested that more than 25% of the motoneurons need to regenerate an axon and successfully innervate a target muscle (Rafuse et al., 1992; Gordon and Tyreman, 2010). A possible mechanism limiting long distance regeneration is the development of a chronically denervated distal nerve after a critical period of 6 to 8 weeks post-lesion. During the protracted long distance regeneration period, denervated Schwann cells gradually fail to support axon regeneration and a non-permissive environment develops.

To investigate the influence of prolonged regeneration time, an identical dox-i-GDNF treatment was performed following avulsion and re-implantation of cervical ventral roots. By performing a root avulsion in the brachial plexus instead of the lumbar plexus, the regeneration distance toward the distal muscles was reduced by half and thus the deleterious effects of chronic denervation were diminished (Figure 1D). We replicated previous observations that timed dox-i-GDNF treatment leads to sustained motoneuron survival and a twofold increase in motor axon regeneration. In addition, timed GDNF treatment leads to enhanced reinnervation of the forelimb paw musculature and recovery of voluntary grip function (Eggers et al., 2019a). The first signs of improved muscle reinnervation were observed before the critical state of chronic denervation has fully developed, demonstrating that beneficial effects of timed GDNF-gene therapy are more robust if target muscle reinnervation can occur within a relatively short time window post-lesion. This further suggests it is erroneous to assume that interventions that were shown to be successful in short distance regeneration models will also be effective in long distance regeneration or in human patients. These observations support the value and the necessity of chronic denervation and long-distance regeneration models in studies on nerve regeneration.

As a first step toward improving distal axonal outgrowth, combined proximal timed dox-i-GDNF gene therapy with a chondroitinase-ABC (ChABC) expression treatment in the distal peripheral nerve (Figure 1E) was performed. Inhibitory chondroitin sulfate proteoglycans (CSPG) accumulate throughout the extracellular matrix of the chronically denervated peripheral nerve and form a major obstacle for regenerating axons. Successful digestion of the inhibitory CSPG sidechains occurs in the distal stump using LV-ChABC (Eggers et al., 2020). Despite successful CSPG digestion and a modest electrophysiological improvement after 45 weeks, distal regeneration was not significantly improved after ChABC treatment and GDNF and ChABC do not display synergistic effects. These results showed that the proximal application of dox-i-GDNF treatment leads to an earlier enhanced electrophysiological response, whereas the distal ChABC treatment effect is modest and occurs during the later stages of the regeneration process.

In summary, the beneficial effect of GDNF gene therapy on motoneuron survival, attenuating motoneuron death up to 1 year is robust and reproducible. Despite increased distal axonal outgrowth, remaining factors that obstruct regeneration and functional recovery still need to be addressed. Here, the chronically denervated Schwann cells and axonal misrouting are considered the primary obstructing factors (Brushart, 1991; Fu and Gordon, 1995; Hoke et al., 2002; Sulaiman and Gordon, 2002; English, 2005; de Ruiter et al., 2008; Sulaiman and Gordon, 2009; O’Daly et al., 2016; Ronchi et al., 2017). A combined treatment strategy aimed at improving motoneuron survival and limiting the deleterious effect of chronic denervation could provide essential support to achieve functional recovery. In the next two sections we discuss the influence of GDNF treatment timing, duration, concentration and location and provide a perspective on future steps to accomplish improved recovery of function.

## Influence of Intervention Timing, Duration, Concentration and Location

**FIGURE 2:**
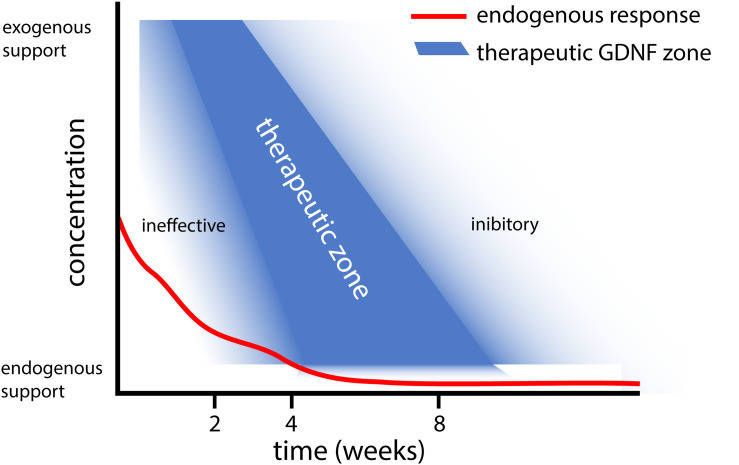
Graphical representation of the proposed relation between GDNF treatment duration and concentration. This graph summarizes our observations that (i) Short and low levels of *endogenous* GDNF expression (red line) is ineffective in achieving motoneuron survival (Eggers et al., 2010), whereas prolonged low dose GDNF results in coil formation (Eggers et al., 2013). (ii) Moderate 4 week timed GDNF expression enhanced motoneuron survival and attenuates coil formation (Eggers et al., 2019a,b). (iii) This contrasts higher GDNF expression levels for 4 weeks, which leads to coil formation (Eggers et al., 2020).

Based on the results obtained with different gene therapy strategies (Figure 1), we propose that there is a relationship between the experimental outcome: (i) GDNF treatment duration and timing, (ii) GDNF concentration, and (iii) location of GDNF delivery. To aid in this discussion, a graphical representation of the proposed relation between GDNF treatment duration, concentration and experimental outcome is depicted in Figure 2.

### GDNF Treatment Timing and Duration

In patients, the lesion severity and possibility of spontaneous recovery needs to be assessed first, which is a challenging task, requires time and delays the decision for surgical repair. Although in experimental studies, delayed surgical repair or GDNF treatment still leads to some motoneuron survival and axonal regeneration, delayed treatment greatly diminishes the degree of motoneuron survival (Wu et al., 2004; Zhou and Wu, 2006). The effect of delayed treatment strongly supports strategies that aim at early neurosurgical repair and GDNF treatment in order for patients to benefit from a maximal degree of axonal outgrowth (Pondaag et al., 2018). Until surgical repair can be performed, bridging the post-lesion period with a systemic pharmacological treatment that delays the acute motoneuron death could result in a more favorable final functional outcome (Nogradi and Vrbova, 2001; Zhang et al., 2005; Hoang et al., 2008; Romeo-Guitart et al., 2017).

In addition to *timing*, the *duration* of GDNF delivery affects the experimental outcome. Delivery of GDNF for a short period results in poor axonal regeneration, whereas prolonged GDNF treatment results in axonal trapping (Marquardt et al., 2015). Our studies extend previous observations where treatment using GDNF protein improved motoneuron survival up to 12 weeks (Li et al., 1995; Yuan et al., 2000; Wu et al., 2003; Bergerot et al., 2004; Zhou and Wu, 2006; Chu et al., 2009, 2012; Pajenda et al., 2014; Ruven et al., 2018). Due to the method used to apply GDNF protein in these studies, the exposure of motoneurons to biologically active GDNF was limited to 2 to 14 days. In these studies, a single topical GDNF protein application, however, is less beneficial for motoneuron survival compared to continued local infusion therapy (Li et al., 1995; Wu et al., 2003; Chu et al., 2009), suggesting a relationship exists between the duration of GDNF treatment and the degree of motoneuron survival. Following 2 weeks of local GDNF protein infusion, the degree of motoneuron survival is identical to our 4 week viral vector-based treatment period (Wu et al., 2003; Eggers et al., 2019b). It is possible that our 4 week timed dox-i-GDNF treatment could be reduced to 2 weeks and still achieve robust motoneuron survival. Based upon our hypothesis of factors influencing the GDNF treatment outcome, however (Figure 2), reducing the treatment duration without adjusting the local GDNF treatment concentration could result in a gradual loss of motoneurons.

### GDNF Treatment Concentration and Motoneuron Survival

Endogenous GDNF expression following ventral root avulsion is elevated for a period of 2 to 4 weeks (Eggers et al., 2010). This is a similar duration compared to above mentioned GDNF protein delivery studies, where frequently a strong reduction of exogenously applied GDNF is observed after 2 weeks (Pajenda et al., 2014). However, only the exogenous application leads to increased motoneuron survival, whereas reimplantation of a ventral root expressing endogenous GDNF prevents motoneuron death for a limited period of 2 weeks (Eggers et al., 2010). An important difference between endogenous and exogenous applied GDNF, is the high local GDNF concentrations that are obtained following local protein application or viral expression. This suggests supraphysiological local GDNF concentrations are essential to achieve successful and prolonged motoneuron survival (Figure 2).

### GDNF Treatment Concentration and Axonal Coil Formation

Regulating the duration of GDNF expression, could potentially overcome axonal coil formation. Although coil formation was attenuated significantly in our first study, with only incidental small isolated coils observed (Eggers et al., 2019b), in a second study increased coil formation was observed in 34% of the dox-i-GDNF treated animals (Eggers et al., 2020). Between these studies, two differences exist which could underlie the observed coil development in the second study. In the study where only small incidental coils were observed, GDNF expression levels were increased 3-fold compared to controls and animals were followed up to 25 weeks post-reimplantation. In contrast, during the first 4 weeks GDNF expression levels were increased 5 fold in the second study where larger coil formation was observed and animals were followed up to 45 weeks post reimplantation.

Axonal outgrowth and coil formation is influenced by the local GDNF concentration (Eggers et al., 2013; Santos et al., 2016; Wang et al., 2018), showing that endogenous or moderate supraphysiological GDNF expression levels for an appropriate period do not result in large axon coils. If, in contrast to moderate GDNF expression, further increasing GDNF concentration during the first 4 weeks stimulates an earlier or advanced state of coil formation, it could be possible that these structures remain present throughout the experiment. Alternatively, it is known that the rtTA transactivator retains some degree of affinity for its DNA binding site, which in the absence of the dox inducer results in low levels of “leaky” expression (Urlinger et al., 2000; Loew et al., 2010; Roney et al., 2016). If local coil formation is the result of continuous GDNF secretion from a leaky vector, based upon Figure 2, there might be a higher chance for the development and observation of coils after a period of 45 weeks rather than after 25 weeks. Although both mechanisms individually or combined could underlie local coil formation, the creation of an immune-evasive rtTA transactivator by fusing with a GlyAla-repeat greatly reduced the leak expression (Hoyng et al., 2014). We were unable to detect leaky expression above endogenous GDNF concentrations using ELISA and immunohistochemical staining, suggesting that leak expression is a less likely cause for residual coil formation.

Our proposed relation between the GDNF concentration, treatment duration and experimental outcome as depicted in Figure 2, suggests that a delicate balance exists to achieve therapeutic GDNF levels for motoneuron survival and axonal outgrowth. Low levels of GDNF appear to not support prolonged motoneuron survival (Eggers et al., 2010), whereas sustained high or low levels of GDNF leads to axonal coil formation (Eggers et al., 2008, 2013). To determine the precise causal mechanism for coil development, more research needs to be performed focusing on GDNF treatment concentration and duration (Santos et al., 2016; Wang et al., 2018).

### Gene Therapy Treatment Location

To support regenerating axons, GDNF treatment in the distal chronically denervated peripheral nerve following ventral root avulsion was unsuccessful in significantly advancing axonal growth and function recovery (Eggers et al., 2013). It is possible that the distal treatment location explains the limited beneficial effect. Distal GDNF expression did not lead to enhanced motoneuron survival, which contrasts proximal GDNF treatment (Eggers et al., 2008, 2019b). A spatially distinct effect of GDNF treatment *in vitro* at the cell body or axon has been shown previously (Zahavi et al., 2015), revealing axon growth and innervation occurred only when GDNF was applied to the axons. As discussed above, increased loss of motoneurons greatly limits the degree of distal axonal outgrowth.

Similarly, although ChABC treatment does not enhance motoneuron survival, our distal ChABC treatment might have been more beneficial when applied more proximally at the reimplantation site (Figure 1E; Eggers et al., 2020). This was shown recently in a study delivering a peptide inhibiting CSPG signaling near the reimplanted nerve root (Li et al., 2015). Increased axonal outgrowth following ChABC treatment is only observed following a transection lesion and not following a crush lesion (Zuo et al., 2002; Muir, 2010; Graham and Muir, 2016). Inhibitory CSPGs are present in the endoneurium surrounding the basal lamina tubes, while the tube itself is relatively permissive. At a transection site, axons will exit the basal lamina tube and are exposed to CSPGs, whereas following a nerve crush axons remain within the pro-regenerative tube. Thus, it is possible that in our avulsion model, most axons enter a basal lamina tube at the implantation site and will not be in close proximity to the distal inhibitory environment, explaining why distal CSPG digestion only has a limited additional effect.

## Perspective on Future Steps to Accomplish Recovery of Function

In future studies, keeping the distal nerve in a pro-regenerative state should be the next priority. The key factors responsible for obstructed axonal regeneration in the peripheral nerve are the loss of neurotrophic support (Funakoshi et al., 1993; Hoke et al., 2002; Eggers et al., 2010), fragmentation of the Schwann cell basal lamina (Brushart et al., 2013) and increased deposits of inhibitory matrix molecules such as CSPGs in the nerve fascicle (Zuo et al., 1998, 2002; Muir, 2010; Graham and Muir, 2016). The unifying component between these growth-promoting and inhibitory factors is the loss of repair Schwann cells. Keeping these cells in a repair phenotype state could therefore be an effective strategy to promote axonal outgrowth (Jessen and Arthur-Farraj, 2019). This requires the ability to specifically target all denervated Schwann cells and to introduce one or multiple factors that are able to achieve this.

Overexpression of a transcription factor could result in a wide range of downstream pro-regenerative molecular changes. As an important regulator in the Schwann cell injury response, the transcription factor c-Jun is such a promising candidate for targeted therapeutic intervention (Parkinson et al., 2008; Arthur-Farraj et al., 2012; Jessen and Mirsky, 2016; Huang et al., 2019). C-Jun has a central role in to promoting expression of the repair program and absence of c-Jun results in the failure of axon growth, functional recovery and neural death (Arthur-Farraj et al., 2012). Recent findings show that moderate c-Jun overexpression levels are beneficial, but supraphysiological levels of c-Jun perturbs myelination (Fazal et al., 2017). Similar to our GDNF treatments, we therefore expect that c-Jun application for therapeutic purposes requires a tightly controlled treatment approach (Huang et al., 2019). We have shown the advantages and potency of viral vector mediated gene therapy and the ability to regulate gene expression. With the development of our immune-evasive stealth gene switch, the gene therapy system was further improved, rendering it even safer. For future research in which factors such as GDNF or C-Jun are applied, it remains to be determined whether the potential low levels of leak expression are detrimental for the treatment strategy or whether the required level of control is sufficient.

To prevent unwanted side effects, gene therapy provides us with the ability to target specific areas or cells with a high degree of precision. LV vectors outperform AAV vectors in transducing Schwann cells in the rat peripheral nerve (Hoyng et al., 2015). However, LV vectors integrate their genetic material in the host cell genome and this could potentially interfere with the function of cellular genes. Adeno-associated viral vectors are increasingly regarded as safe and are well-tolerated following application to the human brain. AAV2 and 8 transduce primate and rat Schwann cells (Girard et al., 2005; Homs et al., 2011). As a first step toward optimizing AAV-mediated gene transfer to Schwann cells we performed a screen of all 9 common serotypes and showed that AAV2 vectors outperform other serotypes in transducing Schwann cells in human peripheral nerve explants, whereas several AAV serotypes efficiently transduced rat Schwann cells (Hoyng et al., 2015). In future studies we will build on these findings and investigate the use of AAV vectors in peripheral nerve repair paradigms.

To keep the peripheral nerve in a pro-regenerative state, ideally, all denervated Schwann cells between the motoneuron and denervated muscle are precisely and equally targeted. This poses a technical challenge, as surgically injecting the entire nerve length including all its thin terminal branches is highly invasive, leads to unwanted additional nerve damage, whereas small diameter nerves are impossible to inject. The recent generation of vector capsids that following intravenous administration can selectively pass the blood brain barrier and transduce neurons located in the brain, spinal cord and DRG is a promising new development (Chan et al., 2017). It is conceivable that comparable viral vectors will be developed that are able to selectively transduce all Schwann cells along an injured peripheral nerve using non-invasive intravenous delivery. When combined with a promotor specific for denervated Schwann cells, this would create the ultimate viral vector, which allows for non-invasive, cell specific, precise control of therapeutic gene expression along the entire denervated peripheral nerve.

## Conclusion

Gene therapy is a powerful tool to improve motoneuron survival and axonal regeneration, and advancements are being made to bring this treatment strategy closer to clinical application. To achieve long distance axonal regeneration, control over treatment location, timing and dose is, however, required. Combined, our data provide a basis to better understand this delicate balance. Although all treatment strategies will need to be tailored to individual patients, ultimately, this and future research could lead to a guiding template which aids the nerve surgeon in selecting the additional gene therapy treatment strategy.

## Author Contributions

RE, FW, MT, MM, and JV wrote this manuscript, conceived, designed, performed, and analyzed the studies on which Figures 1, 2 are based. All authors contributed to the article and approved the submitted version.

## Conflict of Interest

The authors declare that the research was conducted in the absence of any commercial or financial relationships that could be construed as a potential conflict of interest.
